# Secondary structure effects on internal proton transfer in poly-peptides

**DOI:** 10.1063/4.0000003

**Published:** 2020-03-17

**Authors:** M. Bouakil, F. Chirot, M. Girod, P. Dugourd, L. MacAleese

**Affiliations:** 1 Univ Lyon, Université Claude Bernard Lyon 1, CNRS, Institut Lumière Matière, F-69622 Lyon, France; 2 Univ Lyon, Université Claude Bernard Lyon 1, CNRS, Institut des Sciences Analytiques, F-69622 Lyon, France

## Abstract

A pump–probe approach was designed to determine the internal proton transfer (PT) rate in a series of poly-peptide radical cations containing both histidine and tryptophan. The proton transfer is driven by the gas-phase basicity difference between residues. The fragmentation scheme indicates that the gas-phase basicity of histidine is lower than that of radical tryptophan so that histidine is always pulling the proton away from tryptophan. However, the proton transfer requires the two basic sites to be in close proximity, which is rate limited by the peptide conformational dynamics. PT rate measurements were used to probe and explore the peptide conformational dynamics in several poly-glycines/prolines/alanines. For small and unstructured peptides, the PT rate decreases with the size, as expected from a statistical point of view in a flat conformational space. Conversely, if structured conformations are accessible, the structural flexibility of the peptide is decreased. This slows down the occurrence of conformations favorable to proton transfer. A dramatic decrease in the PT rates was observed for peptides HA_n_W, when n changes from 5 to 6. This is attributed to the onset of a stable helix for n = 6. No such discontinuity is observed for poly-glycines or poly-prolines. In HA_n_W, the gas-phase basicity and helix propensity compete for the position of the charge. Interestingly, in this competition between PT and helix formation in HA_6_W, the energy gain associated with helix formation is large enough to slow down the PT beyond experimental time but does not ultimately prevail over the proton preference for histidine.

## INTRODUCTION

Complex molecular systems, such as biomolecules or molecular machines, derive a large part of their remarkable properties from their ability to self-organize and adapt their structure to their environment. Conformational changes in such systems can be triggered by a variety of *stimuli*, including light absorption and temperature or pH changes. They are often accompanied by charge (proton and/or electron) transfer reactions. The latter may either be the origin of the structural change or be triggered by it. In general, however, the two mechanisms are closely related, in a chicken-and-egg situation. Despite its fundamental interest and the potential applications to design smart materials, the problem of understanding the interplay between the two mechanisms is difficult to tackle. Indeed, it involves timescales spanning several orders of magnitude, in relation to the hierarchical structuration of the systems and the coupling between electronic and vibrational degrees of freedom.

Beyond the choice of an observable, one of the key difficulties in studying dynamical processes associated with proton transfer (PT) is the ability to trigger the mechanism in a controlled fashion. One possibility is to use light excitation to form metastable species, which may undergo coupled electron and proton transfer. Some mechanisms are concerted and ultrafast (fs), while others are decoupled, consist of multiple steps, and span longer timescales (ms). Theoretical^1^ and experimental approaches^2–8^ have been proposed to address the steady state energetics and kinetics of, e.g., proton-coupled-electron-transfer (PCET) under different environmental conditions. However, kinetics remains difficult to access directly. Various time-resolved approaches have been applied to investigate in solution the dynamics of such systems,^7,9–15^ ranging from stopped-flow experiments^16^ to transient absorption^17^ or pump–probe experiments using, e.g., ultrafast Raman spectroscopy.^14,15^ However, these studies are usually limited to reduced time ranges, while processes might span more than eight orders of magnitude.^18^ Gas-phase time-resolved photoexcitation dynamics and relaxation in biomolecules have been proposed but are also usually restricted to either ultrafast^19–24^ (<ps) or slow^25^ (>*μ*s) processes although a few recent examples explore the excited state relaxation dynamics over many orders of magnitude in time (ps to ms).^26^ From the structural perspective, ion mobility spectrometry (IMS) comes as a powerful tool to characterize, in the gas phase, the conformation of biomolecules. IMS has been used extensively to study model peptides with, e.g., the formation of α-helices or β-sheets^27,28^ and has more recently evolved toward a standard tool for structural proteomics in the gas phase.^29,30^ Interestingly, internal proton transfer was shown to occur in both model^31^ or biologically relevant peptides^32^ and short oligonucleotides,^33^ with conformational aftermath observable by IMS in each case. However, IMS and optical spectroscopy individually fail to connect experimentally the electronic relaxation dynamics (including charge transfer mechanisms) with structural analysis and conformation dynamics. Part of the objectives of this study is to set landmarks for the concomitant use of IMS and spectroscopy to characterize dynamic photo-induced processes, which are essential for many biomolecular functions.^34^

**SCHEME 1.**
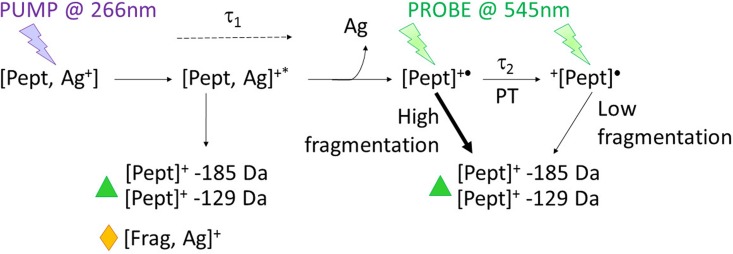
Evolution of the molecular system during the pump–probe experiment: absorption of the UV pump beam by the silver-cationized peptide leads to a photo-excited complex. The latter may evolve back to the ground state and fragment with the charge remaining on silver (yellow diamond). Alternatively, the photo-excited complex can undergo electron transfer and dissociation of neutral silver (τ_1_) leaving the peptide radical system. The latter may also fragment due to excess energy, producing silver-free fragments (green triangle). The surviving radical peptide is driven thermodynamically to its distonic form via a proton transfer (τ_2_). The probe is not absorbed by the ground state complex but is absorbed by radical peptides where the fragmentation ratio depends on the position of the charge.

We have recently shown that, in the model peptide HG_3_W, internal proton transfer and conformational dynamics are deeply connected: conformational dynamics controls the proximity between the basic sites, which limits the kinetics of proton transfer in the peptide.^18^ A gas-phase pump–probe experiment was used, which is based on the coupling between two pulsed lasers and a mass spectrometer (Scheme 1). A UV pump pulse is used to generate a radical peptide cation from [peptide, Ag]^+^ complexes. The mechanism at work is a fast (ps) electron transfer from photo-excited tryptophan to silver, which leads to neutral Ag loss. The optical properties of the resulting radical peptide are then probed by the second laser at 545 nm. At that wavelength, the radical tryptophan cation displays a strong absorption band. As demonstrated earlier, the radical tryptophan cation may decay via an internal proton transfer (PT) into a neutral tryptophan radical, which absorption properties at 545 nm are dramatically attenuated. This difference in absorption properties results in a difference in the photo-fragmentation yield that can be measured by mass spectrometry (MS). This photo-fragmentation yield can be used as a readout of the radical tryptophan cation population. Thus, its evolution, as a function of the delay between the pump and the probe, may be used to characterize the rate of neutralization of tryptophan, which is the PT rate. In the present study, we use the same pump–probe approach to explore the influence of the peptide sequence on the proton transfer dynamics. The photo-fragmentation patterns used to monitor the proton transfer are discussed in the perspective of the mobile proton theory, which is a well-accepted hypothesis in the field of mass spectrometry. The effects of the sequence size and secondary structuration are discussed based on the comparative examination of several peptide families. It confirms the importance of conformational dynamics in the internal proton transfer kinetics.

## METHODS

### Sample preparation

All peptides used (Table S1) were purchased and synthetized by Genecust (Luxembourg) in 10 mg/70% purity batches. Peptides are acetylated and amidated in order to avoid (de-)protonation of the (C-)N-term and control the charge distribution after electrospray ionization (ESI). All peptides are dissolved in water to 300 *μ*M and further diluted, just before ESI, to 10 *μ*M in H_2_O:MeOH (1:1). Silver nitrate (2.5 × 10^−2^ M) was added to the peptide solutions to yield a final concentration of 150 *μ*M. It enables the formation of [peptide, Ag]^+^ complexes by ESI. Due to poor water solubility, poly-alanines were first dissolved (1 mg) in a small volume of trifluoroethanol (TFE):acetonitrile (ACN) (500 *μ*l:210 *μ*l) to which was added 42 *μ*l of a 45 mM solution of silver trifluoroacetate (AgTFA) in ACN. Then, water was progressively added in 100 *μ*l steps to yield a final concentration of 1 mM. The solution was further diluted in H_2_O:ACN (1:1) to 10 *μ*M before ESI. Eventually, the solution was sonicated until clear.

### Mass spectrometry

A commercial linear ion trap mass spectrometer (LTQ-Velos, Thermo Fischer Scientific, San Jose, CA, USA) was used for all experiments. Peptides are ionized with an electrospray (ESI) source with a spray voltage of 5 kV and a sample flow of 5 *μ*l/min. Ions may then be mass selected and fragmented by collisions [collision-induced-dissociation (CID)] or following photo-activation [laser-induced-dissociation (LID)]. Photo-activation of trapped ions in the UV and/or visible ranges is permitted via a fused silica window (3 mm thick, 1 in. diameter) positioned on the axis of the ion traps at the back of the instrument. The central hole of the last ion-trap electrode, toward the window, was enlarged to 5 mm in diameter in order to optimize laser transmission, and ion cloud/laser beams overlap. During photo-activation experiments, collisional activation energy [instrument specific “normalized-collision-energy” (NCE)] is set to 0. Replicates were measured either in the low or high pressure trap (LPT: 3.5 × 10^−4 ^Torr and HPT: 5 × 10^−3 ^Torr). Experimental results (including measured rates) were similar between traps. Thus, they are reported simultaneously.

### Light sources and coupling with MS

**FIG. 1.**
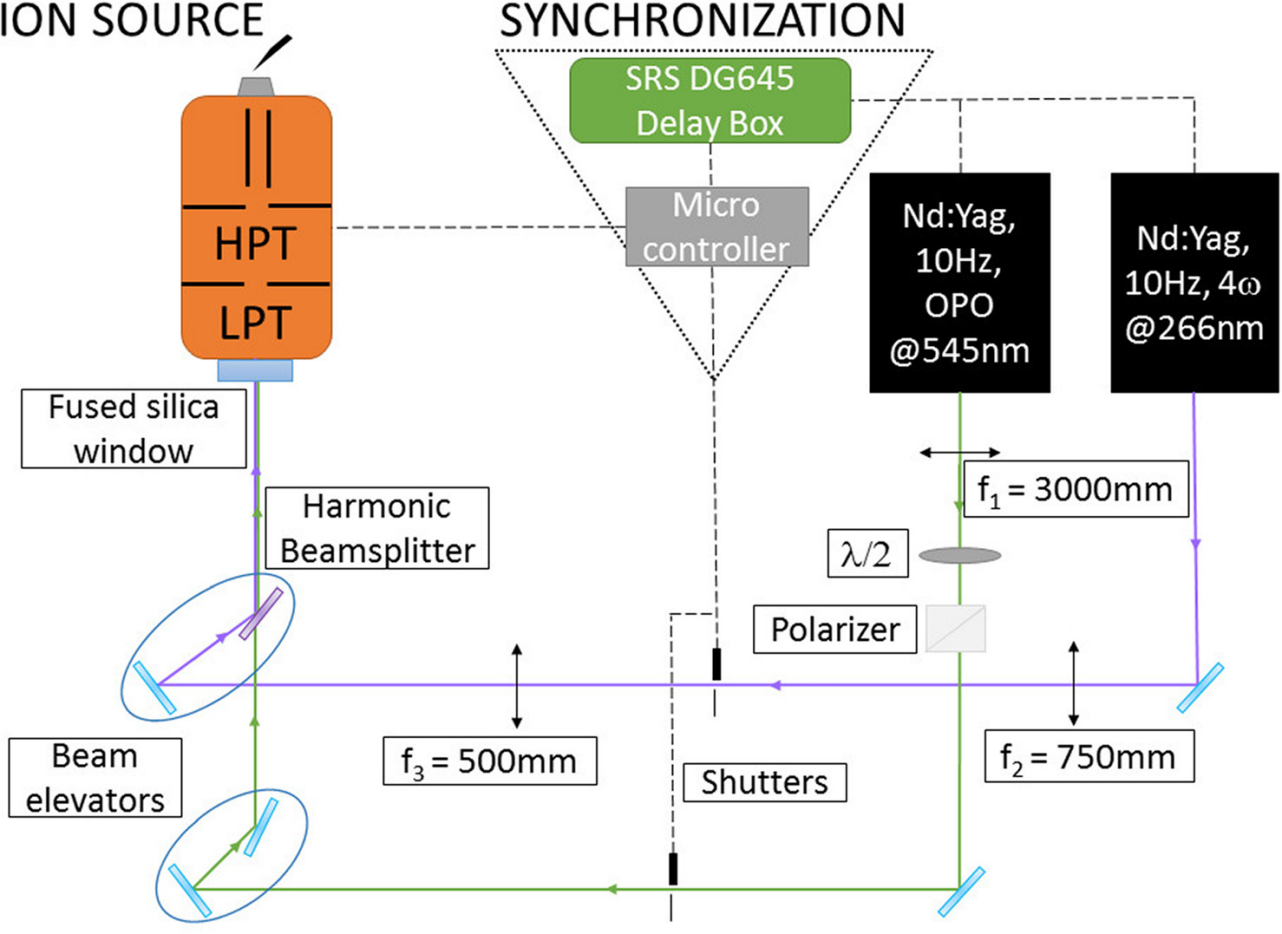
Schematic representation of the experimental setup.

Two laser beams are used, with a pulse width of 5 ns and a repetition rate of 10 Hz: a UV beam at 266 nm (4th harmonic of Nd:YAG-Surelite II, Continuum, Santa Clara, CA, USA) with 2 mJ per pulse and a visible beam at 545 nm (OPO Horizon, pumped by Surelite I, Continuum) with 9 mJ per pulse. For spectroscopy experiments, the wavelength was scanned from 450 nm to 600 nm. Both UV and visible beams are recombined with a harmonic beam splitter (Thorlabs HBSY134) and directed to the ion trap. Both pump and probe beams are triggered externally with a controlled delay using a delay generator (DG645, Stanford Research Systems). The relative arrival times of each laser pulse is controlled with a photodiode at the optical entrance of the mass spectrometer before and after each single series of experiments. The ion trap does not have a constant repetition rate, and thus, a microcontroller (Arduino Uno) is used to trigger the opening of a mechanical shutter (Thorlabs SH05) and allows a single pair of UV and Vis pulses during the presence of selected ions in the trap. A scheme of the setup is shown in Fig. 1.

### Pump–probe experiment sequence

Prior to each experiment, both UV and Vis beams are aligned independently on the maximization of ion photo-fragmentation levels: with [peptide, Ag]^+^ for UV and a reference chromophore ion (QSY7, studied previously^35^) for the 545 nm beam. The UV/Vis overlap is then verified by monitoring at short probe delays (500 ns) the probe-induced photo-fragmentation of the radical peptides produced by the pump (see Fig. S1). Then, the pump–probe experiment starts (see Scheme 1 and Fig. S2) with the mass selection (isolation width 5 *m/z*) and trapping for 170 ms of the parent ion [peptide, Ag]^+^ formed by ESI. During this time, a single pair of UV (pump)–Vis (probe) pulses are allowed in the trap, after which a mass spectrum is recorded. For each pump–probe delay, 120 mass spectra are recorded. A list of up to 200 pump–probe delays was constructed with logarithmic spacing between 50 ns and 20 ms, to which five negative delays were added for control. Acquisitions are performed randomly alternating delays in the list.

### Data analysis

The radical peptide photo-fragmentation ratio at 545 nm (${FR}_{R}^{\mathrm{Vis}}$) is used to evaluate the optical properties of the radical peptide. It is defined [see Eq. (1)] by the ratio of the intensity of fragments of the radical cation produced by the probe ($I_{F}^{\mathrm{Vis}}$) to the original intensity of the parent radical cation [approximated by the sum of $I_{F}^{\mathrm{Vis}}$ with the intensity of the remaining radical peptide ions ($I_{R}$)],

$${FR}_{R}^{\mathrm{Vis}}=\frac{I_{F}^{\mathrm{Vis}}}{I_{F}^{\mathrm{Vis}}+I_{R}}.$$ (1)

However, the pump beam in the UV also generates fragments (see Scheme 1): either before electron transfer, which results in ions containing silver, or after electron transfer to silver, which results in silver-free ions comparable to radical fragments induced by the probe. The measured $I_{F}$ has to be corrected for the contribution of the UV pulse $I_{F}^{UV}$ in order to yield $I_{F}^{\mathrm{Vis}}$. Interestingly, it is possible to differentiate silver-free fragments from other silver-containing fragments based on their isotope pattern and thus specifically quantify them ($I_{F}$ vs $I_{\mathrm{FAg}}$). Additionally, it was found that the pump (UV) forms a constant ratio of silver-free to silver-containing ions ($I_{F}^{UV}/I_{\mathrm{FAg}}=\alpha$), which can be evaluated from mass spectra recorded at negative delays for each set of experiments. Thus, it is possible to correct the total silver-free fragment intensity ($I_{F}$) from the pump-induced offset ($I_{F}^{UV}$) in order to yield the probe-only radical-specific fragments ($I_{F}^{\mathrm{Vis}}$). This correction is only valid if silver-containing fragments cannot be formed by the probe, which is always true after dissociation of the [peptide^+•^, Ag^0^] complex. This leads to the following expression of the radical peptide photo-fragmentation ratio at 545 nm, which can thus be calculated for each mass spectrum,

$${FR}_{R}^{\mathrm{Vis}}=\frac{I_{F}-{\alpha I}_{\mathrm{FAg}}}{I_{R}+I_{F}-{\alpha I}_{\mathrm{FAg}}}=1-\frac{I_{R}}{I_{R}+I_{F}-{\;\alpha I}_{\mathrm{FAg}}}.$$ (2)

For each peptide and each individual mass spectrum, ${FR}_{R}^{\mathrm{Vis}}$ is calculated following Eq. (2) with $I_{F}$ calculated from the two major silver-free fragments observed: loss of 129 Da and 185 Da. For each delay t, the mean ${FR}_{R}^{\mathrm{Vis}}(t)$ (and associated standard deviation of the mean) is calculated from all 120 mass spectra repeats. The set of fragmentation ratios acquired at varying delays is ultimately fitted for delays above 30 *μ*s with the following mono-exponential model functions:

$${FR}_{R}(t>30\;\mu s)=\alpha -\beta .\left(1-e^{-t/\tau_{2}}\right),$$ (3)

where $\alpha ,\;\;\beta ,\;\text{and}\;\tau_{2}$ are adjustable parameters corresponding to an offset in the initial post-pump fragmentation level (α), an amplitude factor (β), and the time constant ($\tau_{2}$), respectively. In the discussion, we focus on the time constant, $\tau_{2}$. For each dataset, fits were performed with the python package lmfit^36^ on resampled data (10^4^ draws). The result for each dataset corresponds to the mean and standard deviation of the resulting 10^4^ optimized time constants (bootstrap approach). Additionally, multiple repeats were performed for each peptide on different days, and the reported time constants and standard deviation (σ) correspond to the weighted average (1/σ^2^) of these repeats. Error bars in plots are set at 2σ (i.e., uncertainty window 95.4%).

**FIG. 2.**
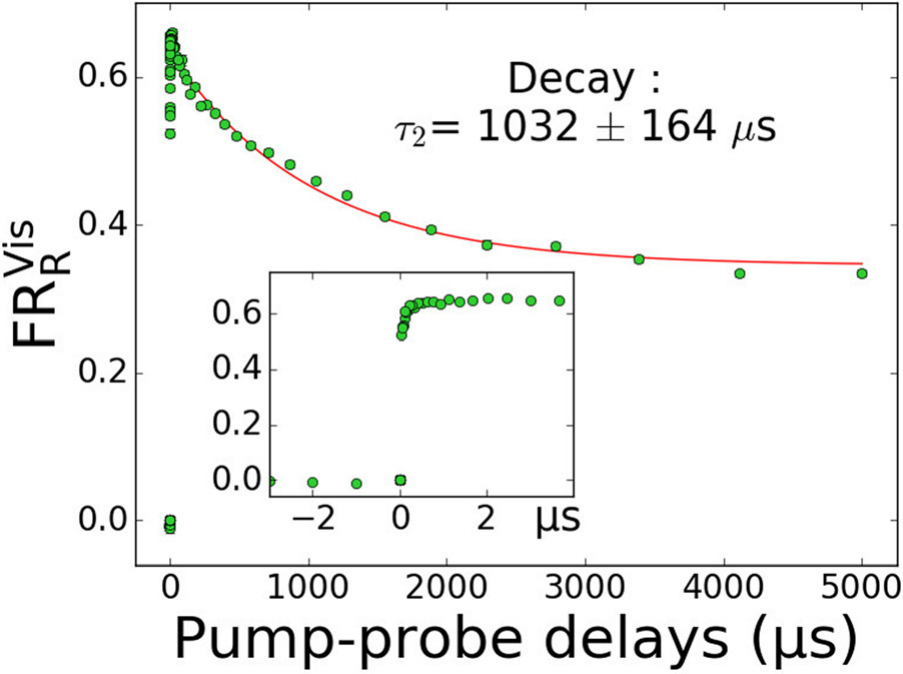
Evolution of the fragmentation ratio of the peptide radical cation [HA_3_W]^+•^ as a function of the pump–probe delay. The experimental data (with correction from the UV offset) is fitted with a mono-exponential decay function for delays >30 *μ*s (red line). The uncertainty window is set to 2σ. The inset displays a zoom on microsecond delays showing a dynamic evolution that may be associated with the neutral silver loss.

Figure S2 displays typical mass spectra obtained, in the case of peptide HA_3_W, along the successive steps of the pump–probe experiment (including controls). Figure 2 displays the evolution of the above-defined fragmentation ratio ${FR}_{R}^{\mathrm{Vis}}$ for the HA_3_W peptide. The full red line shows the result of a fit obtained using the mono-exponential function in Eq. (3). The inset shows the evolution of ${FR}_{R}^{\mathrm{Vis}}$ at delays below 30 *μ*s.

### Ion mobility spectrometry (IMS)

Complementary IMS measurements were performed on both silver-cationized and protonated HA_6_W peptides. The device used is a custom-made tandem ion mobility-mass spectrometry instrument.^37^ IMS measurements were performed in helium (4 Torr) at room temperature, and the drift length was 79 cm. In order to measure the collision cross section (CCS), the arrival time of the ions was measured for different drift voltages ranging from 150 V to 600 V. The CCS was then extracted based on the Mason–Schamp relation,^38^ yielding an error of 2% on the absolute CCS.

## RESULTS AND DISCUSSION

### Proton transfer from radical tryptophan to histidine: Spontaneous but accelerated by the probe

**FIG. 3.**
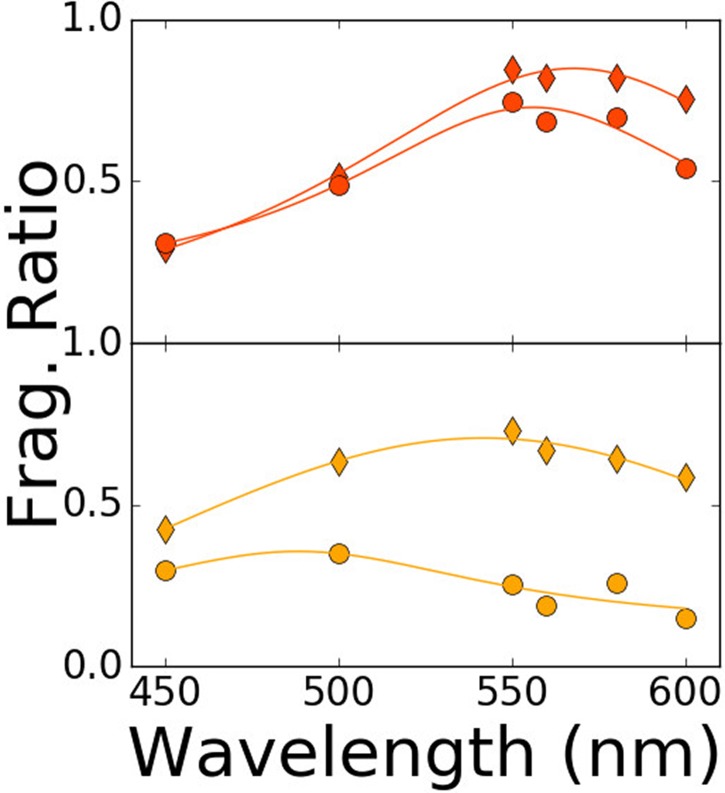
Optical photo-fragmentation spectra of two radical peptides: G_3_W (top frame) and HG_3_W (bottom frame), at different pump–probe delay times: 30 ns (diamond) and 10 ms (circle). Lorentzian lineshapes are plotted to guide the eye.

The 266 nm pump photons resonantly excite the π–π^*^ transition on tryptophan. Similar excitation is followed by relaxation via a dissociative charge-transfer state, leading to H-atom loss in protonated tryptophan^39^ and tryptamine.^40^ The systematic observation of neutral Ag loss in the case of photo-excited [tryptophan-containing peptide, Ag^+^] complexes is assumed to proceed via a similar excited state coupling. It is remarkable that the collisional activation (CID) of these complexes does not lead to any charge transfer between the peptide and the metal:^41,42^ it is an excited state process. The electron transfer from tryptophan to silver is rapid (3.5 ps for HG_3_W^18^) and yields, after neutral Ag-loss, the radical peptide cation. Both the radical and the charge are initially co-localized on tryptophan since the electron excited by the UV pulse was originating from the indole π-system. The optical photo-fragmentation spectra of mass selected radical peptide G_3_W (Fig. 3, top) confirm this hypothesis since they display, independently of the pump–probe delay, the optical signature of the radical cation of tryptophan in the visible range.^43^ However, the presence of histidine on the sequence (HG_3_W) alters this signature, which shifts, after a few milliseconds (Fig. 3, bottom), to the spectral signature of neutral radical tryptophan.^44^ Histidine, thus, appears as a major driving force toward the deprotonation of tryptophan. This is consistent with the high gas phase basicity of imidazole.^45^ It may also be the reason why histidine is often present in active sites of enzymes where the imidazole ring can bind and release protons in the course of enzymatic reactions.^46^

The deprotonation of tryptophan is also apparent from the examination of the fragmentation patterns. Two major fragmentation paths are observed. First, the radical-induced dissociation of the C_α_–C_β_ bond at the tryptophan residue leads to the indole side chain loss with fragment ions at *m/z* 130 or M-129 (with M the *m/z* of the parent radical). Second, a more complex radical rearrangement leads to the loss of the complete tryptophan-containing C-terminus of the peptide with fragment ions at *m/z* 186 or M-185. These fragmentation schemes are observed by CID and LID of the radical cations but were not observed in the CID of either the protonated peptides or the [peptide, Ag^+^] complex. Thus, they are signatures of the fragmentation of the radical species. Both mechanisms were documented and discussed previously, in particular by Siu and co-workers^47^ and Piatkivskyi *et al.*,^48^ and they confirm the presence of π-radical tryptophan. The charge partitioning between fragments is interesting to analyze: M-129 Da and M-185 Da fragment ions are observed exclusively when histidine is present. However, their complementary fragment ions at *m/z* 130 and *m/z* 186 are observed exclusively for peptides without histidine. This provides evidence that histidine modifies the charge partitioning on the peptide fragments via the deprotonation the tryptophan side chain. Since the charge partitioning in such peptide fragments is strongly directed by the relative proton affinity of the two separating moieties,^47,49^ it suggests that the proton affinity of radical tryptophan is, similar to neutral tryptophan [223.9 kcal/mol (Ref. 45)], smaller than that of the histidine imidazole chain [231.5 kcal/mol (Ref. 45)].

Histidine, with its remarkably high gas phase basicity, plays an important role in the mobile proton model.^50,51^ The mobile proton model proposes that protons move from their initial (stable) site upon activation (usually collisional heating). The hypothesis is that the increase in ion internal energy enables protons to overcome barriers and reach less basic sites. Charge-driven fragmentation paths may then be opened even from site where protons are not the most stable from pure proton affinity considerations. In the current study, the radical ion is formed in a metastable conformation, with co-localized radical and charge sites. It is hence thermodynamically driven to the distonic ion. Thus, there is no need *a priori* to invoke the mobile proton model. However, this model contributes to explain why the fragmentation pattern is independent from the delay, i.e., from the progress of the spontaneous PT from tryptophan to histidine. Indeed, fragmentation in the visible range (probe) progressively increases the ion internal energy.^35^ The spontaneous PT is thus accelerated by the probe and leads eventually to the appearance of ground state “thermal” fragments with the proton localized on its energetically preferred site at any delay.

### Evolution at short delays (<30 *μ*s): Dissociation of the metastable complex [peptide^+•^, Ag^0^]

For all systems examined here, the fragmentation ratio is zero for negative probe delays. This is expected since at negative delays, the radical species—which is the only chromophore that absorbs in the visible range—does not exist yet. Thus, in this case, only pump fragmentation is observed [which is corrected for, see Eq. (2)]. It is nevertheless striking that, for most of the peptides examined here, the fragmentation ratio displays an unexpected growth on microsecond timescales, prior to the expected decay (Fig. 2). Importantly, the timescale of these initial features is orders of magnitude larger than the laser jitter—which was measured below one nanosecond—or the pump and probe pulses convolution width—which was estimated below 15 ns. Thus, it is a real feature that reveals a dynamic process with consequences on the observed fragmentation ratio. The radical is expected to be formed rapidly, on picosecond or shorter timescales.^18^ Thus, either its absorption properties are modified or its fragmentation is temporarily quenched.

Both histidine and tryptophan have a strong affinity for the silver cation (respective binding energies 18 kcal/mol and 14.5 kcal/mol above glycine^52^). It is expected that silver binds preferentially to histidine, although silver may bind both simultaneously, as it was shown by ion mobility spectrometry in the case of HG_3_W.^53^ It is hypothesized that, immediately after the electron transfer, the peptide radical cation and neutral silver remain bound in a transient metastable complex. Although different from binding to Ag^0^, binding energies ∼10 kcal/mol have been reported^54^ between histidine/tryptophan and neutral silver in Ag_2_, which is significantly lower (factor 5–10) than binding energies with Ag^+^.^55^ Thus, the neutral silver complex may have a significant lifetime in the gas phase—although the mass detection occurring up to 130 ms after the pump cannot assess it.

While the metastable complex is not dissociated, the probe interacts with a system, which differs from the targeted radical peptide. Thus, many plausible mechanisms could explain the evolution (growth or even decay) of calculated ${FR}_{R}^{\mathrm{Vis}}$ toward the absorption and fragmentation properties of the radical peptide: kinetic energy release via the neutral Ag loss, back-electron transfer from silver to the radical, etc. The presence of the metastable complex may, thus, involve a lower fragmentation efficiency or lead to the overestimation of background fragments generated by UV. In any case, these features, which occur over several hundred nanoseconds, are likely to be associated with the lifetime of the metastable complex. It may interestingly provide insights into the binding strength between silver and the cation, but it reaches beyond the questions addressed in the current study.

### Evolution at longer delays: Proton transfer kinetics

**FIG. 4.**
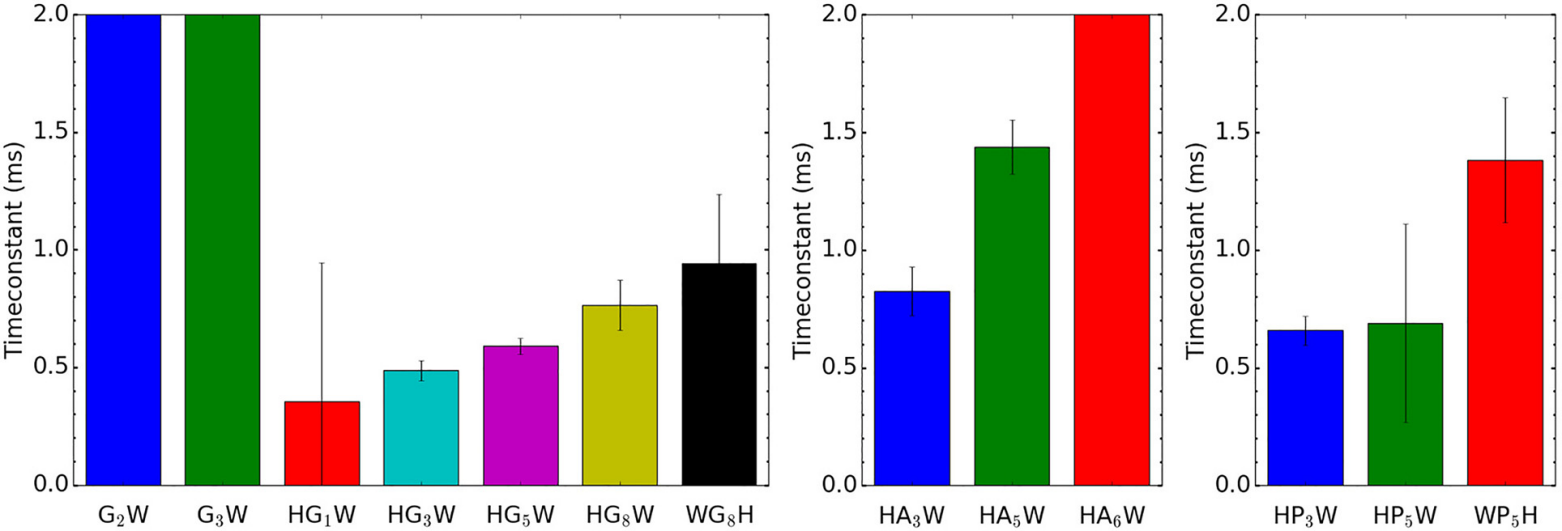
Evolution of the PT time constant in poly-glycines (left), poly-prolines (middle), and poly-alanines (right). Error bars represent 2σ uncertainty windows (95.4%).

For most of the peptides, the initial microsecond step is followed by a slow decay on millisecond timescales. We attribute this decay to the proton transfer from tryptophan to histidine. This is supported by the absence of such a decay in control experiments with peptides missing a histidine residue (G_2_W and G_3_W, see Fig. 4, left, and associated plot in Fig. S3). Remarkably, exponential decays do not tend to the pre-pump level. This is expected since the absorption cross section at 545 nm of the end species (the distonic ion resulting from the proton transfer) is not zero, in contrast to the initial silver complex.

Table I lists time constants determined by mono-exponential fits for delays above 30 *μ*s. These values can be examined by categories of the peptide (poly-glycines, poly-prolines, or poly-alanines) in order to identify trends. When visualizing the evolution of decay time τ_2_ for poly-glycines (see Fig. 4, left panel), the sequence length dependence stands out: the longer the peptide, the less favorable the proton transfer. It may be understood from a purely statistical point of view: while the number of residues increases, the probability that histidine and tryptophan come close enough, and with a favorable orientation to enable proton transfer, decreases. In poly-glycines, increasing the sequence size thus seems to result essentially in an entropic effect: the conformational landscape increases in dimensions, which kinetically slows down the proton transfer. This is true for poly-glycines, which are flexible peptides, i.e., where no specific conformation is particularly energetically favored. In that case, the sequence order (HG_8_W vs WG_8_H) does not seem to play a major role.

**TABLE I.** List of proton transfer time constants and standard deviations extracted from mono-exponential fits on the fragmentation ratio for several families of peptides.

Family	System	Mean values		Individual replicates	
		τ_2_ (*μ*s)	σ (*μ*s)	τ_2_ (*μ*s)	σ (*μ*s)
Controls	G_2_W	>5000	…	…	…
				…	…
	G_3_W	>5000	…	…	…
				…	…
Penta-peptides	HGAGW	704	27	1041	215
				699	27
	HGIGW	634	24	634	24
	HGLGW	615	25	589	30
				681	47
Poly-glycin**es**	HG_1_W	356	295	356	295
	HG_3_W	487	21	563	38
				482	74
				448	35
				478	56
				411	64
	HG_5_W	591	17	513	26
				674	40
				628	46
				723	59
				521	56
				673	53
	HG_8_W	763	53	1139	348
				775	71
				726	83
				1336	539
	WG_8_H	942	147	1199	707
				853	228
				990	200
Poly-alanines	HA_3_W	825	52	662	106
				700	88
				1032	82
	HA_5_W	1438	58	1184	339
				1744	190
				1604	94
				1262	84
	HA_6_W	>5000	…	…	…
				…	…
				…	…
				…	…
Poly-prolines	HP_3_W	657	31	449	52
				570	100
				770	43
				1266	159
	HP_5_W	690	212	646	589
				588	236
				1921	896
				2371	2001
	WP_5_H	1383	133	1374	158
				1575	411
				1313	303

**FIG. 5.**
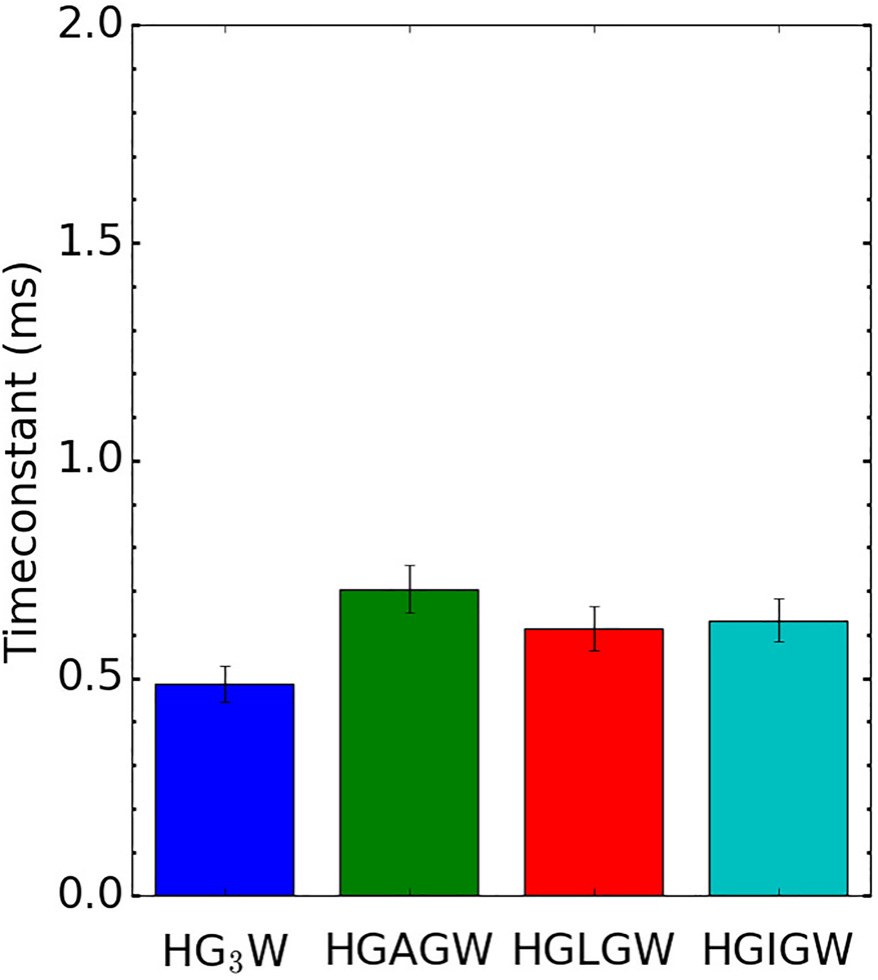
Evolution of the PT time constant on a series of penta-peptides with modification of the amino acid in the middle of the sequence.

The same trend with the peptide size is also observed for small poly-prolines (Fig. 4, middle panel), similar to the unstructured poly-glycines. This trend is also observed for poly-alanines up to five alanines (see Fig. 4, right panel). It is remarkable, however, that time constants are significantly larger compared to poly-glycines with the same number of residues. This increase may be due to a lower flexibility of poly-alanines and poly-prolines vs poly-glycines for reasons of steric hindrance of the side chains. It was however observed that single amino acid modifications hardly affect the overall PT time constant (see Fig. 5). Thus, in terms of peptide sequence composition, collective effects seem to affect more importantly the peptide flexibility than the presence of any particular individual amino acid.

Remarkably, however, the addition of just one more alanine to form HA_6_W results in a considerable decrease in the PT rate (i.e., increase of the PT time constant) such that it cannot be quantified experimentally within the experimental boundaries (see Fig. S4). This slowing down of the proton transfer rate beyond measured times (>5 ms) cannot be explained only by the entropy factor. Another important parameter must bias the conformational dynamics against proton transfer. An interesting hypothesis is the occurrence of secondary structuration in poly-alanines. Indeed, alanines are among the most stabilizing residues for the formation of α-helices in solution.^56^ Additionally, Kaleta and Jarrold^57^ and Hudgins *et al.*^58^ reported on the propensity of poly-alanines of seven or more residues to form α-/π-helices in the gas phase. Interestingly, this matches the size of poly-alanines at which a drastic PT rate modification was observed in the current study. Additionally, one can expect that histidine and tryptophan do not disrupt the formation of the secondary structure. Indeed, Kaleta and Jarrold also showed that the insertion in poly-alanines of other residues with lower helix propensity, such as glycine or lysine, and, in particular at terminal positions, may not disrupt completely the helical conformation.^57^

Formation of a helix as a stable secondary structure is, hence, likely in the case of HA_6_W. It would correspond to the appearance of a potential energy (enthalpy) well in the otherwise rather flat conformational space. This would consequently rigidify the peptide structure and hamper any reactive contact between histidine and tryptophan. The question is whether this structure pre-exists in the silver complex. Ion mobility spectrometry (IMS) experiments were performed and reveal that both protonated and silver-cationized HA_6_W peptides have a very close collisional cross section (CCS, 208 Å^2^ and 211 Å^2^, respectively) and hence most likely very close structures. HA_6_W is thought to adopt a random globular conformation to maximize the solvation of the charge (proton) on histidine, similar to poly-alanines with a lysine residue at the N-term studied by Hudgins and Jarrold.^59^ Thus, since the silver complex has a nearly identical CCS with the protonated peptide, it is also most likely non-helical. The radical peptide generated after electron transfer to silver in this unstructured complex is, likewise, initially unstructured. However, the charge localized on tryptophan, i.e., at the C-term, forms a supplementary stabilizing factor for the helical structures since the positive charge interacts favorably with the helix dipole.^59^ Once formed, the geometry prevents the formation of reactive conformations. Thus, there must be a competition between proton transfer and formation of a stabilizing helical structure. This type of competition was observed before by Kinnear and co-workers in poly-valines.^60^ In these poly-valines, the energy gained from stabilizing a helix over-compensate the energy loss associated with proton localization at a less basic site (C-term). Similarly, it seems plausible that the appearance of secondary structuration in competition with proton transfer is the element that principally affects the conformational dynamics in HA_6_W, slowing down the proton transfer away from tryptophan. Remarkably, the exclusive observation of deprotonated radical tryptophan fragments, as stated above, advocates in favor of competing although still energetically disfavored helix formation in HA_6_W.

## CONCLUSION

A pump–probe approach was designed to monitor the evolution of the internal proton transfer rate in a series of poly-peptides. The proton transfer was earlier found to be rate limited by the peptide conformational dynamics, and the rationale was to use it as a signature for the very same peptide conformational dynamics. With this tool, various poly-peptide families such as poly-glycines, poly-prolines, and poly-alanines with varying lengths were examined and compared. Interestingly, dynamic features could be detected at timescales around or below 1 μs, which may be attributed to the kinetics of dissociation of neutral Ag from its metastable complex formed with the radical peptide immediately after electron transfer. Although beyond the scope of this particular study, the interaction between metal atoms with oxidation state 0 and biomolecules is yet a relatively unexplored field, which may become of interest in the context of the generalization of metal nanoparticles for bio-imaging and bio-medical applications.

However, the main objectives were to identify trends in the peptide conformational dynamics along two directions: the sequence length and sequence composition. Generally, the larger the peptide, the larger the conformational space and the smaller the PT rate. Thus, the conformational dynamics is governed by entropy for small and non-structured peptides. From a pure gas-phase basicity point of view, the PT from radical tryptophan to histidine is spontaneous and thermodynamically driven. Thus, it should always occur although rate-limited by conformational dynamics. Actually, it should always occur unless the formation of a secondary structure, such as a helix, overcompensates the energy cost of keeping the proton on the tryptophan radical. Since the PT rate in HA_6_W is slowed down beyond measurable times, can we conclude that the proton transfer is disfavored due to the formation of a helix? According to the mobile proton theory, heating should favor fragmentation with the proton moving to less basic sites. The presence of histidine on the sequence is shown to result exclusively in photo-fragments associated with deprotonated tryptophan for all examined peptides. Thus, for all the systems examined, the proton transfer to histidine seems to yield a significantly more stable thermodynamic minimum. This also stands for HA_6_W. It seems that, thus, for HA_6_W, the energy gain brought by helix formation is large enough to considerably bias the exploration of the conformational space and induce a strong competition between PT and helix formation; but it is not sufficient to fully compensate the gas-phase basicity difference between histidine and tryptophan. This is consistent with the statement by Jarrold and co-workers that poly-alanines start to be helical in the gas-phase from seven successive residues. Our results also concur in the presence of a secondary structuration onset between six and seven alanines.

These results emphasize the functional role of structural dynamics in biological machines where charge transfer mechanisms may be controlled by secondary structuration. These questions are fundamental in biology and also find very pragmatic applications due to the rapid development of opto-genetic tools, mainly inspired by natural photoreceptor modules, which require an increasing level of control on photo-activation mechanisms. The coupling between time-resolved spectroscopy and ion mobility is the natural perspective for this work in order to support structural hypothesis.

## SUPPLEMENTARY MATERIAL

See the supplementary material for the details of all peptides examined. A graphical representation of the experimental sequence is illustrated with mass spectra in the case of the peptide HA_3_W. The control of the probe alignment is also illustrated in the case of the peptide HA_3_W. Negative controls are provided (absence of PT decay in the case of the histidine-free peptide G_3_W). The very slow decays observed in the case of HA_6_W are also shown.

## References

[1] A. Migliore , N. F. Polizzi , M. J. Therien , and D. N. Beratan , Chem. Rev. 114, 3381 (2014). 10.1021/cr4006654 24684625 PMC4317057

[2] D. W. Mulder , M. W. Ratzloff , M. Bruschi , C. Greco , E. Koonce , J. W. Peters , and P. W. King , J. Am. Chem. Soc. 136, 15394 (2014). 10.1021/ja508629m 25286239

[3] S. J. Nara , L. Valgimigli , G. F. Pedulli , and D. A. Pratt , J. Am. Chem. Soc. 132, 863 (2010). 10.1021/ja907921w 20000763

[4] S. Prashanthi and P. R. Bangal , Chem. Commun. 2009, 1757 10.1039/b818892k 19294286

[5] P. H. Kumar , Y. Venkatesh , S. Prashanthi , D. Siva , B. Ramakrishna , and P. R. Bangal , Phys. Chem. Chem. Phys. 16, 23173 (2014). 10.1039/C4CP02505A 25253044

[6] J. D. Megiatto, Jr. , D. D. Mendez-Hernandez , M. E. Tejeda-Ferrari , A.-L. Teillout , M. J. Llansola-Portoles , G. Kodis , O. G. Poluektov , T. Rajh , V. Mujica , T. L. Groy , D. Gust , T. A. Moore , and A. L. Moore , Nat. Chem. 6, 423 (2014). 10.1038/nchem.1862 24755594

[7] L. M. Oltrogge , Q. Wang , and S. G. Boxer , Biochemistry 53, 5947 (2014). 10.1021/bi500147n 25184668 PMC4172208

[8] T. J. Meyer , M. H. V. Huynh , and H. H. Thorp , Angew. Chem., Int. Ed. 46, 5284 (2007). 10.1002/anie.200600917 17604381

[9] S. Y. Reece , M. R. Seyedsayamdost , J. Stubbe , and D. G. Nocera , J. Am. Chem. Soc. 129, 8500 (2007). 10.1021/ja0704434 17567129

[10] T. Irebo , S. Y. Reece , M. Sjödin , D. G. Nocera , and L. Hammarström , J. Am. Chem. Soc. 129, 15462 (2007). 10.1021/ja073012u 18027937

[11] J. M. Hodgkiss , N. H. Damrauer , S. Pressé , J. Rosenthal , and D. G. Nocera , J. Phys. Chem. B 110, 18853 (2006). 10.1021/jp056703q 16986876

[12] M. M. Warren , M. Kaucikas , A. Fitzpatrick , P. Champion , J. T. Sage , and J. J. van Thor , Nat. Commun. 4, 1461 (2013). 10.1038/ncomms2460 23403562

[13] J. M. Hodgkiss , A. Krivokapić , and D. G. Nocera , J. Phys. Chem. B 111, 8258 (2007). 10.1021/jp070447v 17590036

[14] R. Du , C. Liu , Y. Zhao , K.-M. Pei , H.-G. Wang , X. Zheng , M. Li , J.-D. Xue , and D. L. Phillips , J. Phys. Chem. B 115, 8266 (2011). 10.1021/jp203185a 21615104

[15] C. Fang , R. R. Frontiera , R. Tran , and R. A. Mathies , Nature 462, 200 (2009). 10.1038/nature08527 19907490

[16] V. V. Smirnov and J. P. Roth , J. Biol. Inorg. Chem. 19, 1137 (2014). 10.1007/s00775-014-1169-7 25023856

[17] M.-T. Zhang and L. Hammarström , J. Am. Chem. Soc. 133, 8806 (2011). 10.1021/ja201536b 21500853

[18] L. MacAleese , S. Hermelin , K. El Hage , P. Chouzenoux , A. Kulesza , R. Antoine , L. Bonacina , M. Meuwly , J.-P. Wolf , and P. Dugourd , J. Am. Chem. Soc. 138, 4401 (2016). 10.1021/jacs.5b12587 26974184

[19] L. Guyon , T. Tabarin , B. Thuillier , R. Antoine , E. Brunner , V. Boutou , J.-P. Wolf , and P. Dugourd , J. Chem. Phys. 128, 75103 (2008). 10.1063/1.2828558 18298175

[20] G. Reitsma , O. Gonzalez-Magaña , O. Versolato , M. Door , R. Hoekstra , E. Suraud , B. Fischer , N. Camus , M. Kremer , R. Moshammer , and T. Schlathölter , Int. J. Mass Spectrom. 365-366, 365 (2014). 10.1016/j.ijms.2014.01.004

[21] D. Nolting , T. Schultz , I. V. Hertel , and R. Weinkauf , Phys. Chem. Chem. Phys. 8, 5247 (2006). 10.1039/b609726j 17203149

[22] D. Nolting , R. Weinkauf , I. V. Hertel , and T. Schultz , ChemPhysChem 8, 751 (2007). 10.1002/cphc.200600727 17366508

[23] S. Soorkia , M. Broquier , and G. Grégoire , J. Phys. Chem. Lett. 5, 4349 (2014). 10.1021/jz502387q 26273986

[24] A. S. Chatterley , C. W. West , V. G. Stavros , and J. R. R. Verlet , Chem. Sci. 5, 3963 (2014). 10.1039/C4SC01493F 26274076

[25] K. Støchkel , J. A. Wyer , M.-B. S. Kirketerp , and S. Brøndsted Nielsen , J. Am. Soc. Mass Spectrom. 21, 1884 (2010). 10.1016/j.jasms.2010.07.004 20696594

[26] S. Soorkia , M. Broquier , and G. Grégoire , Phys. Chem. Chem. Phys. 18, 23785 (2016). 10.1039/C6CP04050K 27524459

[27] M. F. Jarrold , Phys. Chem. Chem. Phys. 9, 1659 (2007). 10.1039/b612615d 17396176

[28] A. E. Counterman and D. E. Clemmer , J. Phys. Chem. B 108, 4885 (2004). 10.1021/jp036454a 40533934

[29] C. Uetrecht , R. J. Rose , E. van Duijn , K. Lorenzen , and A. J. R. Heck , Chem. Soc. Rev. 39, 1633 (2010). 10.1039/B914002F 20419213

[30] E. G. Marklund , M. T. Degiacomi , C. V. Robinson , A. J. Baldwin , and J. L. P. Benesch , Structure 23, 791 (2015). 10.1016/j.str.2015.02.010 25800554

[31] M. Kohtani , T. C. Jones , R. Sudha , and M. F. Jarrold , J. Am. Chem. Soc. 128, 7193 (2006). 10.1021/ja056745s 16734471

[32] J. Li , W. Lyu , G. Rossetti , A. Konijnenberg , A. Natalello , E. Ippoliti , M. Orozco , F. Sobott , R. Grandori , and P. Carloni , J. Phys. Chem. Lett. 8, 1105 (2017). 10.1021/acs.jpclett.7b00127 28207277

[33] A. Arcella , J. Dreyer , E. Ippoliti , I. Ivani , G. Portella , V. Gabelica , P. Carloni , and M. Orozco , Angew. Chem., Int. Ed. 54, 467–471 (2014). 10.1002/anie.201406910 25417598

[34] J. M. Christie , L. Blackwood , J. Petersen , and S. Sullivan , Plant Cell Physiol. 56, 401 (2015). 10.1093/pcp/pcu196 25516569 PMC4357641

[35] M. Bouakil , A. Kulesza , S. Daly , L. MacAleese , R. Antoine , and P. Dugourd , J. Am. Soc. Mass Spectrom. 28, 2181 (2017). 10.1007/s13361-017-1733-9 28755260 PMC5594054

[36] M. Newville , R. Otten , A. Nelson , A. Ingargiola , T. Stensitzki , D. Allan , A. Fox , F. Carter , Michał , D. Pustakhod , Y. Ram , Glenn , C. Deil , Stuermer , A. Beelen , O. Frost , N. Zobrist , G. Pasquevich , A. L. R. Hansen , T. Spillane , S. Caldwell , A. Polloreno , A. Hannum , J. Zimmermann , J. Borreguero , J. Fraine , deep-42-thought, B. F. Maier , B. Gamari , and A. Almarza (2019). “Imfit/Imfit-py,” Zenodo, V.1.0.0, Dataset. 10.5281/zenodo.598352.

[37] A.-L. Simon , F. Chirot , C. M. Choi , C. Clavier , M. Barbaire , J. Maurelli , X. Dagany , L. MacAleese , and P. Dugourd , Rev. Sci. Instrum. 86, 094101 (2015). 10.1063/1.4930604 26429458

[38] H. E. Revercomb and E. A. Mason , Anal. Chem. 47, 970 (1975). 10.1021/ac60357a043

[39] H. Kang , C. Dedonder-Lardeux , C. Jouvet , S. Martrenchard , G. Grégoire , C. Desfrançois , J.-P. Schermann , M. Barat , and J. A. Fayeton , Phys. Chem. Chem. Phys. 6, 2628 (2004). 10.1039/B315425D 19785164

[40] H. Kang , C. Jouvet , C. Dedonder-Lardeux , S. Martrenchard , C. Charrière , G. Grégoire , C. Desfrançois , J. P. Schermann , M. Barat , and J. A. Fayeton , J. Chem. Phys. 122, 084307 (2005). 10.1063/1.1851503 15836039

[41] L. Feketeová , M. W. Wong , and R. A. J. O'Hair , Eur. Phys. J. D 60, 11 (2010). 10.1140/epjd/e2010-00019-6

[42] T. Shoeib , J. Zhao , H. E. Aribi , A. C. Hopkinson , and K. W. Michael Siu , J. Am. Soc. Mass Spectrom. 24, 38 (2013). 10.1007/s13361-012-0511-y 23238948

[43] B. Bellina , I. Compagnon , S. Houver , P. Maître , A.-R. Allouche , R. Antoine , and P. Dugourd , Angew. Chem., Int. Ed. 50, 11430 (2011). 10.1002/anie.201104783 21987502

[44] L. Joly , R. Antoine , A.-R. Allouche , and P. Dugourd , J. Am. Chem. Soc. 130, 13832 (2008). 10.1021/ja804508d 18817390

[45] A. G. Harrison , Mass Spectrom. Rev. 16, 201 (1997). 10.1002/(SICI)1098-2787(1997)16:4<201::AID-MAS3>3.0.CO;2-L

[46] F. Schneider , Angew. Chem., Int. Ed. Engl. 17, 583 (1978). 10.1002/anie.197805831 101098

[47] C.-K. Siu , Y. Ke , G. Orlova , A. C. Hopkinson , and K. W. M. Siu , J. Am. Soc. Mass Spectrom. 19, 1799 (2008). 10.1016/j.jasms.2008.09.026 18930412

[48] A. Piatkivskyi , J. K.-C. Lau , G. Berden , J. Oomens , A. C. Hopkinson , K. M. Siu , and V. Ryzhov , Eur. J. Mass Spectrom. 25, 112 (2019). 10.1177/1469066718802547 30282467

[49] S. A. McLuckey , D. Cameron , and R. G. Cooks , J. Am. Chem. Soc. 103, 1313 (1981). 10.1021/ja00396a001

[50] V. H. Wysocki , G. Tsaprailis , L. L. Smith , and L. A. Breci , J. Mass Spectrom. 35, 1399 (2000). 10.1002/1096-9888(200012)35:12<1399::AID-JMS86>3.0.CO;2-R 11180630

[51] B. Paizs and S. Suhai , Mass Spectrom. Rev. 24, 508 (2005). 10.1002/mas.20024 15389847

[52] V. W.-M. Lee , H. Li , T.-C. Lau , R. Guevremont , and K. W. Michael Siu , J. Am. Soc. Mass Spectrom. 9, 760 (1998). 10.1016/S1044-0305(98)00051-8

[53] B. Bellina , I. Compagnon , L. MacAleese , F. Chirot , J. Lemoine , P. Maître , M. Broyer , R. Antoine , A. Kulesza , R. Mitrić , V. Bonačić-Koutecký , and P. Dugourd , Phys. Chem. Chem. Phys. 14, 11433 (2012). 10.1039/c2cp40924k 22801489

[54] A. A. Buglak , R. R. Ramazanov , and A. I. Kononov , Amino Acids 51, 855 (2019). 10.1007/s00726-019-02728-z 30900086

[55] J. Jover , R. Bosque , and J. Sales , Dalton Trans. 2008, 6441 10.1039/b805860a 19002332

[56] C. Nick Pace and J. Martin Scholtz , Biophys. J. 75, 422 (1998). 10.1016/S0006-3495(98)77529-0 9649402 PMC1299714

[57] D. T. Kaleta and M. F. Jarrold , J. Phys. Chem. B 105, 4436 (2001). 10.1021/jp004446d

[58] R. R. Hudgins , M. A. Ratner , and M. F. Jarrold , J. Am. Chem. Soc. 120, 12974 (1998). 10.1021/ja983021q

[59] R. R. Hudgins and M. F. Jarrold , J. Am. Chem. Soc. 121, 3494 (1999). 10.1021/ja983996a

[60] B. S. Kinnear , D. T. Kaleta , M. Kohtani , R. R. Hudgins , and M. F. Jarrold , J. Am. Chem. Soc. 122, 9243 (2000). 10.1021/ja001207v

